# Peptide Vaccine Therapy in Colorectal Cancer

**DOI:** 10.3390/vaccines1010001

**Published:** 2012-08-23

**Authors:** Aleksandra Bartnik, Ajit Johnson Nirmal, Shi-Yu Yang

**Affiliations:** Research Department of General Surgery, Division of Surgery and Interventional Science, UCL Medical School, University College London, Royal Free Campus, Rowland Hill Street, London NW3 2PF, UK; E-Mails: o.bartnik@ucl.ac.uk (A.B.); ajit.nirmal.10@alumni.ucl.ac.uk (A.J.N.)

**Keywords:** peptide vaccine therapy, colorectal cancer, EphA2, survivin, SART3, CEA and MUC-1

## Abstract

Colorectal cancer is the third most common cause of cancer-related deaths and the second most prevalent (after breast cancer) in the western world. High metastatic relapse rates and severe side effects associated with the adjuvant treatment have urged oncologists and clinicians to find a novel, less toxic therapeutic strategy. Considering the limited success of the past clinical trials involving peptide vaccine therapy to treat colorectal cancer, it is necessary to revise our knowledge of the immune system and its potential use in tackling cancer. This review presents the efforts of the scientific community in the development of peptide vaccine therapy for colorectal cancer. We review recent clinical trials and the strategies for immunologic monitoring of responses to peptide vaccine therapy. We also discuss the mechanisms underlying the therapy and potential molecular targets in colon cancer.

## 1. Introduction

Colorectal cancer (CRC) is the third most common cause of cancer-related deaths among men and women in the western world [1,2]. Europe has the highest incidence of CRC with an estimated 412,900 new cases in 2006 [1] closely followed by the USA with 145,290 colorectal cancers in 2008 [3]. With a prevalence of around 2.4 million cases, CRC ranks as the second most prevalent, after breast cancer. The discrepancy between incidence and prevalence reflects high rates of survival due to early detection and effective management [4]. Treatment with curative intent invariably involves surgical resection for stages I-III, followed by 5-fluorouracil-based adjuvant therapy for stages II–III. The latter is considered an essential part of the treatment due to the high metastatic relapse rates (75%) within 3 years after resection [5]. Although the adjuvant treatment substantially improves survival, it is associated with side effects that affect overall health and the quality of life [6]. Therefore it is necessary to develop a novel, less toxic adjuvant therapy to treat the disease, especially disseminated CRC.

Since the discovery of tumour antigens in 1991, immunotherapy has become a potential alternative to conventional chemotherapy [7]. In 2010, immunotherapy in colon cancer started to become a reality with several clinical trials involving peptide vaccine therapy underway. Here we review recent advancements in the development of peptide vaccine therapy, including the clinical trials, and discuss the mechanisms underlying the therapy and potential molecular targets in colon cancer. The immunologic monitoring of cellular immune responses following peptide vaccine therapy is also discussed. 

## 2. Results and Discussion

### 2.1. The Mechanism of Peptide Vaccine Therapy

Immunotherapy is based on the principle of aberrant expression of proteins by tumours, which either over-express ubiquitous proteins or express proteins that unusually occur in the original tissue of the cancer. Tumour cells that bear these antigens can be distinguished from the normal tissue by the immune system in the same way that bacteria or virus-infected cells are. Recognition of an unusual antigen starts an immune response aimed at elimination of the cell and generation of antigen-specific immune cells that provide long-lasting immunity. However, immune-mediated regression of cancer hardly ever takes place in real life due to the low effectiveness of wild-type antigens (generated by the tumour) in stimulating host immune responses. 

The aim of vaccine therapy is to provide a highly immunogenic antigen, such as peptides, which are capable of stimulating immune system to mount a cytotoxic attack against the tumour cells. The desired effect would be stabilization or even regression of the disease.

### 2.2. The Mechanism of Immune Destruction of the Tumour

Following an inoculation, the circulating peptides are degraded in the circulation and the antigenic products are endocytosed by antigen-presenting cells (APC), which migrate to lymph nodes where they present the antigen in a processed form to T cells. These are at the forefront of the battle against the tumour. There are two main types of T cells according to the surface markers and function. CD8+ T cells, also known as cytotoxic T lymphocytes (CTL), are responsible for direct killing of tumour cells. They induce apoptosis by release of granzymes and perforins or by presenting Fas ligand (FasL) to Fas death receptor on the target cells. Another consequence of granzyme release is target cell necrosis [8]. CD4+ T cells differentiate further upon activation. CD4+ T cells type 1 act as helper cells that secrete cytokines to recruit more CTLs, as well as other members of the innate immune system such as macrophages and natural killer cells. 

The interaction between individual cells is mediated by two major histocompatibility complex (MHC) proteins, MHC classes I and II (also known as HLA I and II), present on the surface of cells. The antigenic protein from the circulation is processed by cellular machinery into a several-amino acid-peptide that can enter the MHC molecule for presentation to T cell receptor (TCR).

**Figure 1:**
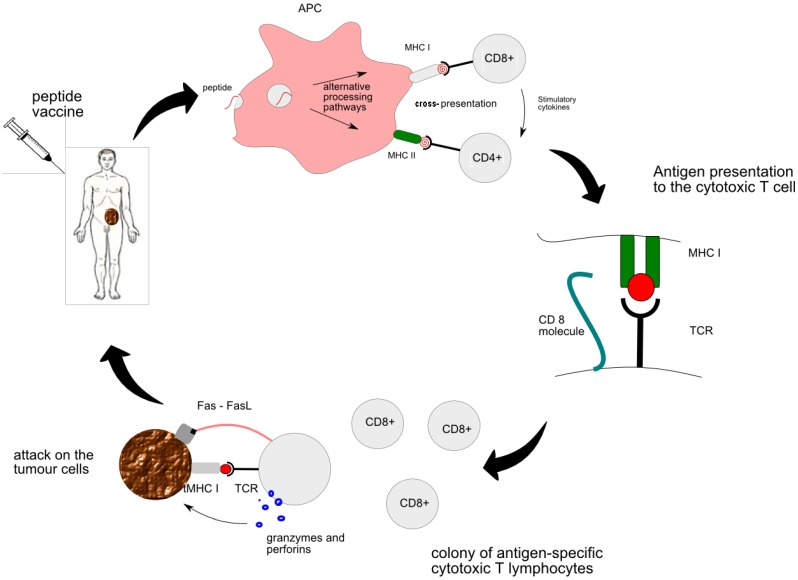
The mechanism of anti-tumour effect of peptide vaccine therapy: introduction of vaccine to the bloodstream; processing and presentation of the peptide by the antigen-presenting cell (APC) in a lymph node resulting in activation of CD4+ helper T cells and CD8+ cytotoxic T cells; interaction between MHC I molecule on APC and T cell receptor (TCR) during antigen presentation facilitated by CD8 molecule; generation of tumour-specific CTLs capable of lysing tumour cells: degranulation of CTL following recognition of tumour antigen and Fas-mediated transduction of death signal to the tumour.

● **MHC class I** is responsible for presentation of the vaccine-derived peptide between APC and naïve CTLs. Primed CTLs are able to recognize the genuine tumour antigen presented by the MHC I on the surface of the tumour. Thus activated CTL sends out a death signal to the tumour.
● **MHC class II** is responsible for “the talk” between APC and CD4+ T cells and subsequent generation of helper T cells. Cytokines secreted by helper T cells, such as IL-2, are essential for activation of strong cytotoxic pathways and potentiation of anti-tumour response [9]. All in all, the CTL encounter with the peptide is at the heart of peptide vaccine therapy. More details of interaction between individual cells can be found in Figure 1.

### 2.3. The Mechanisms whereby Tumour Evades Immune System

Various immune cells are normally found infiltrating the mass of a tumour, some of which are specialized to recognize the tumour-associated antigens. Although there is evidence of a positive correlation between the number of these cells and good prognosis, their effect on the tumour is considered insignificant [10,11]. It has been proposed that tumours are capable of developing ways to evade immune system or down-modulate immune responses [12].

● One such mechanism is expression of FasL, which is a ligand for Fas receptor present on CTL. Fas functions as a death receptor which, upon binding of its ligand expressed on the tumour surface, induces CTL to undergo apoptosis. Additionally, expression of FasL provides a degree of resistance to Fas-induced apoptosis in tumour cells themselves [13].
● Another mechanism involves expression of altered peptide ligands for T cell receptors (TCR) by the tumour. “Altered Peptide Ligand” (APL) are analogs of immunogenic peptides in which the TCR contact sites have been manipulated [14]. Recognition of a peptide ligand by CTL leads to lysis of the tumour cell. However, ligands that are slightly altered retain their ability to bind the TCR but the outcome of this interaction is different. The altered peptides may be related to the agonist ligand on the basis of their structural homology. Thus, partially activating APL is a subset of the antagonists and thereby modulates the activity of CTL [15].
● Oncogenic signalling pathways within tumour cells and immunologic checkpoints in the tumour microenvironment also play a crucial role in promoting immunologic tolerance. For example, tumour releases factors that induce inhibition of both innate and adaptive antitumor immunity. Stat3 activation in tumours, as well as Braf activation, can induce release of factors such as IL10 that induce Stat3 signalling in NK cells, granulocytes, inhibiting their tumouricidal activity. Stat3 is also activated within conventional dendritic cells (CDC) in the tumour, converting them to toleragenic DC, which can induce T cell anergy and possibly regulatory T cells (Treg). Plasmacytoid DC (PDC) or PDC-related cells in the tumour microenvironment upregulate indoleamine 2,3-dioxygenase (IDO), an enzyme that metabolizes tryptophan. T cells are very sensitive to tryptophan depletion. Tumours can express co-inhibitory B7 family members, such as B7-H1 and B7-H4, which downregulate T cell activation and/or cytolytic activity. They can also induce B7-H1 and B7-H4 expression on tumour associated macrophages (TAM). Related immature myeloid cells or myeloid suppressor cells can further inhibit antitumor T cells via production of NO by the enzyme arginase. [16,17].
● Regulatory T cells are an important inhibitor of antitumor immunity. T cell activation in the absence of appropriate co-stimulatory signals leads to T cell anergy and generation of induced regulatory T cells (Treg). Treg, characterized by the FoxP3 transcription factor, upregulate a number of cell membrane molecules, including Lag3, CTLA4, GITR, and neuropilin. Treg can inhibit effector T cell activation and function via T-T inhibition or inhibition of antigen presenting cells [17].
● Finally, cancers avoid recognition by the immune system by means of defective antigen presentation. This can be achieved by reduced expression of HLA type I (MHC I), which is a common event in colorectal cancer [18], as well as, reduced expression of antigen-processing machinery or tumour-associated antigen itself.

Immune-competence of cancer patients who receive chemotherapy is already partially compromised. The design of peptide vaccines is aimed at overcoming the described problems by delivering highly immunogenic antigens combined with adjuvants that stimulate the immune system.

### 2.4. The Ways of Enhancing Immune Response to Peptide Vaccination

Special design of the amino acid sequence of a peptide can enhance the interaction with the TCR. This usually involves creation of a peptide epitope with single amino acid substitutions, which exhibits improved binding to MHC or affinity for the TCR. Such manipulations are capable of potentiating secretion of interleukin 2 (IL-2, cytokine immune system signaling molecule), which is a potent immune-boosting cytokine. Other modifications, involving substitution at the peptide terminals, results in improved bio-stability and reduced degradation by seric proteases [19]. Effectiveness of the vaccine can also be improved by the peptide delivery system. The employment of vectors such as liposomes and dendritic cells with potent antigen-presenting properties has been exploited with success [20]. Use of adjuvants is another way to increase immunogenicity of a peptide. These adjuvants include IL-2 (immune system signaling molecule belonging to cytokine family) which activates CTL, GM-CSF which stimulates APC, as well as incomplete Freund’s adjuvant (IFA) and heat shock protein (hsp) 90 [9]. Novel solutions for adjuvant formulations come from the field of nanotechnology. Poly-γ-glutamic acid adjuvant nanoparticles used in colorectal cancer mouse model have been found to exert immune-boosting effect similar to that of complete Freund’s adjuvant which is considered the strongest adjuvant available. These nanoparticles have the advantage of causing little or no liver and kidney toxicity [21].

### 2.5. Why Peptide Vaccine?

The use of synthetic peptides is a fairly recent endeavour. In the early days of immune therapy tumour antigens were delivered to the patient in a form of tumour lysates. Nowadays tumour lysates are being pulsed into dendritic cells (DC) as a way of facilitating peptide presentation. Another recent development is the use of a retrovirus carrying the antigen-encoding gene, which inserts itself in the host genome leading to production of large quantities of the antigen [22]. Against this background, peptide therapy bears several advantages. Firstly it provides maximal essential component as opposed to tumour lysates where the antigen is diluted in a bulk of biological mass. Secondly, monitoring of immune response is easier with peptide-based therapy as it requires evaluation of only one cytotoxic T lymphocyte (CTL) type, which is specific for the peptide; while tumour lysates carry multiple antigens which make immune monitoring much more complicated. Furthermore, laboratory-based synthesis of peptides offers virtually unlimited possibilities for modifications and design enhancement. Last but not least, the process of generation of peptide vaccine and its use in the clinic is fairly cost-effective. 

The main drawback is the human leukocyte antigen (HLA)-type restricted nature of the therapy. HLA molecules are expressed in a multitude of super-types and individual antigens are only capable of interacting with a particular HLA-super type. In the clinical context this means that only patients carrying the required HLA allele will be able to respond to the therapy. This substantially limits the application of the therapy. Moreover, use of self-antigens carries a risk of inducing autoimmunity. This kind of therapy is also contraindicated in patients with existing autoimmune diseases, where exposure of the over-reactive immune system to a highly immunogenic antigen can have unpredictable consequences. An example of that comes from trials on immune therapy in Parkinson’s disease where the patients’ condition was reported to be aggravated following vaccination against a neuroprotein [22].

### 2.6. Monitoring of Response to Peptide Vaccine Therapy

Two aspects of response to peptide therapy are commonly evaluated in clinical setting: clinical outcome as the primary endpoint and stimulation of cell-mediated toxicity as a secondary endpoint. Several techniques have been used to measure the frequency and activity of antigen-specific CTLs, including ELISPOT assay, flow cytometry, RT-PCR, HLA/epitope tetramer assay. This is often combined with evaluation by means of serum cytokines and chromium (υ¹Cr) release assays, and proliferation studies [23]. The υ¹Cr release assays has a number of disadvantages, including low sensitivity, poor labelling and high spontaneous release of isotope from some tumour target cells.

● ELISPOT assays are used to assess secretion of proteins by CTLs which are indicative of an activated state, such as, INF-γ, granzyme B, IL-2 [24]. The assays are performed using peripheral blood mononuclear cells (PBMC) isolated from patient blood sample and the result is expressed as the number of reactive CTLs per 100,000 PBMC [23].
● Following T-cell receptor recognition of antigenic peptide–MHC class I complexes on the surface of target cells, CTLs induce target cell apoptosis through directed exocytosis of perforin and granzymes. The cytotoxic signalling leads to the activation of the caspase cascade that can be measured using flow cytometer. The assay involved labelling of P8_15_, EL_4_ and T_2_ lymphoma cells with a cell tracker dye DDAO-SE and staining permeabilized cells with antibody against cleaved (activated) caspase-3. This assay proved to be robust and reliable in evaluating antigen-specific CTL [25].
● Another approach is to use flow cytometry for detection of apoptosis in CTL target cells (for example cytomegalovirus bearing appropriate HLA molecule). During apoptosis, phosphatidylserine (PS) is externalized on the surface of the cells and is available for binding of annexin V. Both granule marker and annexin V assays allow evaluation of the cytotoxic potential of tumour-specific CTLs [8].
● By contrast, HLA/epitope tetramer assay is used for mere enumeration of the antigen-specific CTLs. However, when combined with intracellular cytokine staining it gives a complete picture of quantity and function of the TCLs.
● Lastly, RT-PCR has been used to evaluate cytokine gene expression, including INF-gamma and granzyme B, by CTLs [25,26]. Cells are harvesting from patients who have been administered peptide vaccination followed by RNA extracted from it. cDNA is synthesised and RT-PCR analysis are performed using forward and reverse primers for IFNγ or CD8, The synthesised cDNA is further validated by the measurement of Gene expression using ABI Prism 7700 Sequence Detection System.

Ideally the immune response should correlate with the primary endpoints, which reflect the disease progression. Sadly this has been exceptionally rare in the clinical trials so far. In addition to patient survival, the levels of tumour markers are measured using fluorescence-based immunoassays for monitoring of tumour growth and response to therapy [27]. Also, delayed-type hypersensitivity reactions are commonly performed and evaluated for macroscopic changes at the injection site and lymphocytic infiltration of the skin biopsy material [28]. Finally, CT scan has been employed for evaluation of liver metastases [29]. 

### 2.7. Targets for Peptide Vaccine Therapy in Colon Cancer

Tumour self-antigens are broadly categorized according to their specificity for the tumour. Differentiation tumour antigens present in both tumour and its original tissue, but are expressed at higher levels in the tumour. Tumour-specific antigens occur in tumours cells but are absent in normal adult tissues. Tumour/testis antigens are additionally expressed in the gonads. Unique tumour antigens constitute a separate group. These arise from nucleotide deletions or alternative splicing and characterize each single tumour. 

The choice of each one of these targets for vaccine therapy carries its specific risks as well as benefits. Targeting differentiation antigens, owing to their presence outside the tumour, can elicit a degree of adverse reaction in the healthy tissue. An example of that comes from clinical trials in melanoma where use of peptide vaccines directed against proteins involved in melanin biosynthesis leads to development of skin depigmentation (vitiligo). With regards to unique antigens, some evidence speaks in favour of their higher immunogenicity. The immune response against unique antigens has been found to persist for longer following tumour resection compared with shared tumour-specific antigens [30]. However, this notion remains controversial, as the data on unique antigens is scarce. On top of that, identification of unique antigens would require sequencing of the whole genome for every single tumour, which is not feasible in the present day. Nonetheless, a patient-tailored vaccine therapy is an appealing perspective. 

Tumour-specific antigens have been the most popular targets for vaccine therapy in general and in the context of colon cancer in particular. It must be noted that immunogenicity of colorectal cancers is considerably lower compared to the most immunogenic among cancers, *i.e.*, melanoma. Nonetheless, the few antigen-based vaccines that have been developed show the ability to induce antigen-specific CTL and cause reduction in the tumour size in animal studies. Translation of these findings to a clinical setting was not completely successful. 

One particular pattern of response to peptide vaccine therapy prevails across all clinical studies. That is, strong induction of antigen-specific CTLs is not associated with improved clinical outcome. Frequently reported side effects of the therapy are: ulceration at the injection site, fever, fatigue, nausea, anorexia. However, serious adverse reactions are very rare. The protocols commonly require 3 or more injection courses at 2–3 weeks intervals and patients who show satisfactory response are offered continuation of the therapy. It has been argued that the lag between the first jab and the clinically relevant response is too long, especially in the case of advanced cancer patients who may not survive the necessary time. Below is a brief review of selected antigen targets in colon cancer including their biological role, results of *in vivo* studies and clinical trials. The remainder are summarized in the Table 1. 

**Table 1:** Antigen targets in colon cancer, their biological role, results of *in vivo* studies and clinical trials for peptide vaccination.

Peptides	Targets	Mechanism	Type of Study	Results	Side Effects	Comments	Reference
EphA2-derived peptide	EphaA2	EphA2-specific CTL	*In vivo*: colon cancer liver metastasis mouse model	Prevents progression of tumour in the liver	No liver or kidney toxicity	Safe to apply clinically to treat colon cancer liver metastases	[21]
RNF43-721			Phase 1 clinical trial in colorectal cancer			Vaccinations were well tolerated	[31]
ABT-737	Bcl-2 small molecule inhibitor	Inhibition of anti-apoptotic Bcl-2 family	*In vivo*: mouse colon cancer model	Sensitized cancer cells to the antitumor effect of antigen-specific immunotherapy		Improve survival rate	[32]
Multi- peptide cocktail:Epitomes of HER2, MVF, GMP and *n*-MDP	Multiple targets:HER2, MVF, GMP and *n*-MDP	Inhibition of EGF-2	Phase 1 clinical trial in solid cancers including 4 colorectal	25% SD	No serious adverse events, autoimmune disease, or cardiotoxicity		[33]
Endoglin	Endoglin	Inhibition of angiogenesis	CT26 colon carcinoma mouse model	Inhibition of tumour growth and angiogenesis			[34]
CEA_691_	Carcinoembryonic antigen	Induction of tumor-specific CTLs	Colon carcinoma mouse model	CEA-specific CTL responses were augmented Antigen-specific proliferation of splenocytes and secretion of Th1 cytokines increased Survival rate increased		Potential for future clinical applications	[35]
MUC1, MHC class II helper peptides	A cell surface associated protein:Mucin 1	Stimulation of IFN-gamma-producing CD4 (+) helper cells,Induction of CTLs specific to MUC1 and other undefined MC38 tumour antigens	A MUC1-tolerant colon cancer mouse model	In the therapeutic setting, tumour burden was significantly reduced In the prophylactic setting, tumour was completely rejected		Potential for future clinical applications	[36]
CEA_526–533_, NP_52–59_	Carcinoembryonic antigen	Activation of tumor-specific CTLs	Murine colon adenocarcinoma mouse model				
OX40L	TNF family protein		CT26 colon cancer mouse model	Inhibition of tumour growth in a dose and route dependent manner Repression of CRC lung metastasis in a dose dependent manner		Potential use for colon metastasis treatment	[37]
Heat-Shock Protein Gp96	Heat-Shock Protein	Induction of tumour-specific CTLs	Clinical trial in colorectal cancer liver metastases after tumour resection	Induction of colon carcinoma-specific CTLs in 52% patients Two-year overall survival and disease-free survival were significantly improved	No significant toxicity	Possible clinical benefit for CRC liver metastatic patients	[38]
SART3_109–118_ SART3_315–323_	SART	Induction of tumour-specific CTLs	Clinical trial in patients with advanced colorectal cancer	Increased cellular immune responses to the tumour and the vaccinated peptide Dose-dependent responses	No serious adverse events	Encourage further development of SART3 peptide vaccine for colorectal cancer patients	[39]
Lck-derived peptides		Induction of tumor-specific CTLs					[40]
CEA_605–613_ and Flt3L	CEA	Induction of tumor-specific CTLs	Clinical trial metastatic or recurrent colorectal cancer				[41]

### 2.8. EphA2

Ephrin type-A receptor 2 (EphA2) is a member of a large tyrosine kinase receptor family. Eph receptors play an important role in oncogenesis [42] and tumour angiogenesis [43]. It has been found that EphA2 is over-expressed in colorectal carcinomas [44] and other various cancers [45]. The fact that highest level of EphA2 expression is observed in metastatic lesions makes EphA2 a high-priority target for immunotherapy [21]. Its utility as a tumour antigen has been evaluated in a liver metastasis mouse model transfected with EphaA2-positive colon cancer tumour. Immunization-induced antigen-specific CTL was associated with a degree of protection against the tumour expansion. A recent study using EphA2-derived peptide in combination with amphiphilic nanoparticles in a murine model demonstrated high a level of immunity against colorectal cancer liver metastases following immunization. Interestingly the study also found the novel nanoparticle-based adjuvant to be more beneficial in terms of effectiveness and toxicity [21]. 

### 2.9. Survivin

Survivin is a protein which inhibits cancer cell apoptosis, is highly expressed during embryological development, and becomes undetectable in adult tissues [46]. A study of 171 cases of colorectal cancer found its abundant expression in more than 50% with no expression in adjacent normal tissue [47]. One of two variants of the protein, surviving-2B, was found to possess a peptide capable of binding HLA-A24. The peptide induced HLA-A24-restricted cytotoxic T cells, which subsequently exhibited high toxicity against HLA-A24-positive survivin-2B-positive cancer *in vitro* [48]. In 2003, the first clinical trial involving survivin-2B was conducted in advanced and recurrent colorectal cancer patients [24]. The therapy resulted in an increased proportion of peptide-specific CTL in the general population of circulating CTLs from 0.09% to 0.35%. However, this was not accompanied by significantly improved clinical outcome. One out of 17 patients showed minor reduction of tumour size and 6 patients had a reduced CEA marker confined to the duration of the therapy [29,35,48,49,50].

### 2.10. SART3

SART3 is a tumour-rejection antigen which is expressed in more than 70% colorectal cancers but is not present in normal non-malignant tissues [51]. It was one of the first targets for vaccine therapy tested in a setting of a Phase I clinical trial. Myiagi *et al.* used a vaccine formulation combining two SART3 antigenic epitopes recognized by HLA-A24-restricted CTLs with IFA in colorectal cancer patients. Immunization resulted in significant induction of tumour-specific CTL in 7 of 11 patients. Two patients presented with induction of INF-γ following the sixth vaccination time. However, augmentation of cellular immunity was not associated with improved clinical outcome. Likewise, no induction of IgG or IgE specific for the peptides was observed. An adverse reaction at the injection site manifested as itching and redness occurred in 6 patients. The median number of vaccination times was 8 and the median observation period was 5 months. Despite the somewhat promising result no further trials involving SART antigen has been published since 2001 [52].

### 2.11. CEA

Carcinoembryonic antigen (CEA) is a useful tumour marker which is correlated with tumour burden in the mouse metastasis model and has been used to monitor CRC metastasis development [53]. A mouse model for colorectal cancer was used to evaluate its suitability as a target for peptide vaccine therapy alone and in combination with an antibody that mimics a specific CEA epitope. Transgenic mice carrying tumours positive for CEA and HLA-A2 were vaccinated with dendritic cells pulsed with a CTL epitope of CEA. Complete tumour regression was observed in as many as 25% of mice. In the cured mice, no tumour recurrence was observed for 90 days. Use of the antibody leads to antigen presentation by both MHC I and II, whereby it stimulates CD4+ helper cells. The use of CEA-mimicking antibody was associated with production of INF-γ by CD4+, which is thought to improve the expression of MHC class I by the tumour. This approach resulted in increased proliferation and cytokine-production by tumour-specific CTLs and a complete sustained regression of the tumour in 67.5% mice. 

Overall, immunization with CEA resulted in enhanced immune response and survival. Further improvement in both endpoints was seen with the combination therapy. This constitutes an interesting case for employment of both antibody-mediated and cell-mediated immunity to achieve greater anti-tumour response. It also consolidates the importance of T helper cells in stimulation of cytotoxic killing of the tumour cells [54]. 

### 2.12. MUC-1

MUC-1 is an epithelial membrane antigen, which bound to the apical membrane of the secretory cells. It has been found that MUC-1 is expressed in more than 70% colorectal cancers and correlates with poor prognosis [55]. An early clinical trial involving mucin-like peptides was conducted in 1996 with unremarkable results and no further clinical investigations followed. However, recent animal experiments using MUC1 peptide-based vaccine therapy yielded promising results [36]. The researchers used a cocktail of strong adjuvants: MHC class II-restricted pan helper peptide, unmethylated CpG oligodeoxynucleotide, and GM-CSF. In addition to stimulating antigen-specific CTL, the vaccine elicited abundant secretion of INF-gamma by activated helper T cell type 1. This resulted in significant reduction of an established tumour. Interestingly, the vaccination was completely successful in preventing growth of a tumour transfected after immunization. This opens a window for the use of the therapy in the prophylaxis of colorectal cancer [28,36]. 

We have discussed some of the selected antigen targets in colon cancer including their biological role, results of *in vivo* studies and clinical trials. The remainder are summarized in the Table 1.

## 3. Conclusions

Development of a novel, less toxic therapeutic strategy to treat advanced and disseminated colorectal cancer has been a goal for oncologists and clinicians for many years. The discovery of tumour antigens in 1991 has opened a window for the use of immune therapy in the treatment of cancer. In the last two decades, a great deal of effort has been made to identify the molecular targets for immune therapy in colorectal cancer; some of those targets proved effective in a pre-clinical setting. Moreover, the immune therapy has been shown to cause minimal side effects in a clinical setting, which makes it a desirable alternative to the current systemic treatments. Its ability to induce strong and specific anti-tumour immune responses in patients has been well documented using the current immunologic monitoring techniques. However, these findings did not correlate with improved clinical outcome. This calls for more accurate strategies for immune monitoring and could become the next direction for research.
